# The Interface Between Empirical and Simulation-Based Ground-Motion Models

**DOI:** 10.1007/s00024-018-2044-1

**Published:** 2018-11-20

**Authors:** Gail Marie Atkinson

**Affiliations:** Department of Earth Sciences, Western University, London, N6A 5B7 Canada

**Keywords:** Ground-motion models, PSHA, empirical model, seismological model

## Abstract

Ground-motion models (GMMs) are a key driver for the results of probabilistic seismic hazard analyses and their uncertainty. GMMs that bridge seismological and empirical approaches are an effective tool to represent the distribution of ground motion and its uncertainty in seismic hazard assessment. A methodology is presented that uses ground-motion data recorded at seismograph sites in eastern North America and shows how they can be used to calibrate simple scalable seismological models of ground-motion generation and propagation. Such GMMs can directly account for the gross features of source scaling (magnitude and stress parameter), attenuation, site response, and kappa effects. It is shown that, by application of appropriate GMM strategies, sigma (aleatory uncertainty) could be greatly reduced, resulting in lower calculated hazard for nuclear plants founded on rock. This reduction in sigma requires that high-quality seismic monitoring (e.g., broadband seismograph stations) be installed and operated over a period of years (in addition to strong-motion stations), and that an ongoing investment be made in data analysis and targeted GMM development using the data.

## Introduction

Ground-motion models (GMMs), also referred to as ground-motion prediction equations, are a key component in probabilistic seismic hazard analysis (PSHA), and often the most important uncertainty affecting PSHA results. GMMs provide median estimates of ground-motion amplitudes as a function of explanatory variables such as magnitude, distance, and site conditions, along with estimates of variability. Empirical GMMs are commonly used in data-rich regions such as California and Japan; For instance, the second-generation Pacific Earthquake Engineering Research–Next Generation Attenuation-West (NGA-W2) project (Bozorgnia et al. 2014) includes empirical GMMs for crustal earthquakes in active tectonic regions (Abrahamson et al. 2014; Boore et al. 2014; Campbell and Bozorgnia 2014; Chiou and Youngs 2014; Idriss 2014), and is widely used in practice (e.g., Petersen et al. 2015).

An alternative method, commonly used in data-poor regions, is to derive a GMM using a simulation-based approach, in which a seismological model is calibrated with a set of empirical data. The advantage of such an approach is that robust magnitude and distance scaling behaviors can be imposed, whilst accommodating regional features that can be determined from limited available data. There are numerous examples of such simulation-based models in practice, including stochastic point-source models (Boore 1983; Atkinson and Boore 1995; Toro et al. 1997; Boore 2003) and finite-source stochastic and broadband simulations (Beresnev and Atkinson 1997; Motazedian and Atkinson 2005; Assatourians and Atkinson 2007; Frankel 2009, 2015). Note that, for stochastic models, simulations are not always required, as a random process statistical model can also be applied.

**Fig. 1:**
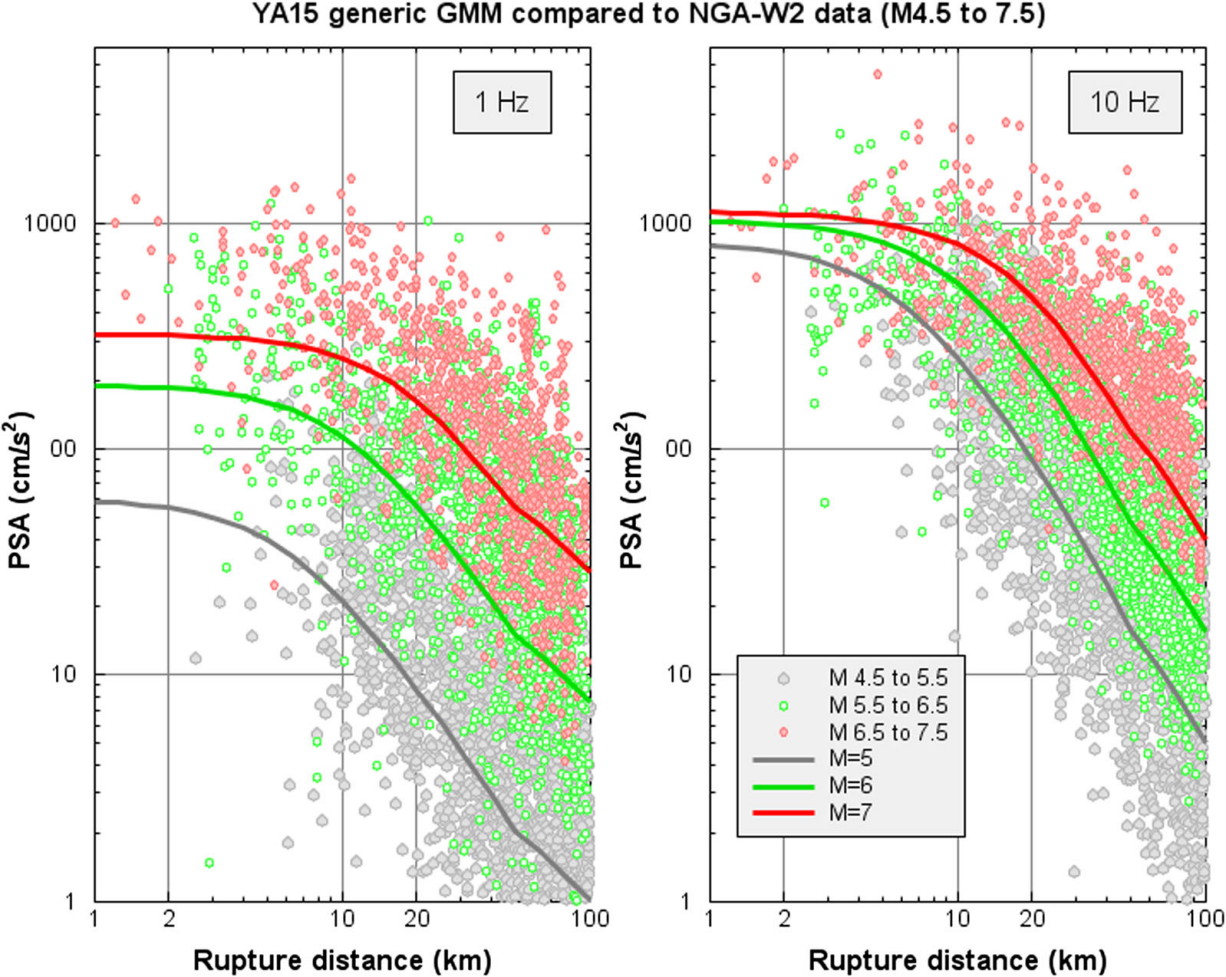
Comparison of YA15 generic GMM for *B*/*C* site conditions for crustal earthquakes in active regions, developed using equivalent point-source approach (lines) with ground-motion amplitudes of NGA-W2 database, corrected to equivalent values for *B*/*C* sites using the empirical site model of Seyhan and Stewart (2014) (circles). Magnitude ranges are color-coded

Yenier and Atkinson (2015a, b) developed a regionally adjustable generic GMM based on the concept of equivalent point-source simulations. They derived a robust simulation-based GMM that can be adjusted to different regions by modifying the seismological input parameters (e.g., geometrical spreading, stress parameter, and calibration factor models) and examined the applicability of the model for earthquakes in California and central and eastern North America (Yenier and Atkinson 2015b). The parameters for the generic GMM were originally defined by calibrating a seismological model to match the empirical ground-motion amplitudes recorded in California (Yenier and Atkinson 2015a). Specifically, Yenier and Atkinson (2015a) used the rich California ground-motion database to define elements of the functional form and calibrate the overall model scaling behavior in magnitude and distance. They also determined the geometric spreading (including a term to model the effects of near-distance saturation), anelastic attenuation, and stress parameter models that describe ground-motion amplitudes for California. The model was parametrized in a way that isolates the effects of magnitude scaling, stress parameter scaling, geometrical spreading, and anelastic attenuation on ground-motion amplitudes, so that the approach can be readily transported to other regions by modifying just a few regional source and attenuation parameters. Figure 1 illustrates the YA15 (Yenier and Atkinson 2015a) equivalent point-source model GMM for California and active crustal regions (for *B*/*C* site conditions of 760 m/s), in comparison with the underlying NGA-W2 data used in model development. The generic GMM matches the data as well as strictly empirical GMMs developed from the NGA-W2 database and has the added benefit of being parameterized by simple seismological parameters.

## The Generic Ground-Motion Model: A Calibrated Equivalent Point-Source Approach

Atkinson et al. (2015) used the generic equivalent point-source approach of Yenier and Atkinson (YA15, Fig. 1) to develop a GMM for ground motions on rock sites in eastern North America (ENA). The model can be written as follows, with model coefficients being provided in the “Appendix.” The equation is used for the geometric mean horizontal component of motion in natural log units as a function of closest distance to the rupture (*D*_rup_):

1 $$\ln \left( Y \right) = F_{\text{E}} + F_{\text{Z}} + \gamma D_{\text{rup }} + F_{\text{S}} + C,$$

where ln(*Y*) is the (natural) logarithm of a ground-motion intensity measure, such as peak ground acceleration (PGA), velocity (PGV), and 5 %-damped pseudospectral acceleration (PSA) at a selected oscillator frequency. *F*_E_, *F*_Z_, and *F*_S_ are the model components for earthquake source, geometrical spreading, and site amplification, respectively. The anelastic attenuation (*γ*) and empirical calibration (*C*) coefficients are frequency dependent. The *C* term is an empirical constant that scales the simulation amplitudes to match the amplitude of the observations. The source (*F*_E_) and geometrical spreading (*F*_Z_, including near-distance saturation) terms are constrained in their scaling behavior by the equivalent point-source simulations that were validated using the rich empirical database from California (e.g., Fig. 1). Regional ground-motion data for ENA were inverted to determine the anelastic attenuation coefficients (*γ*), site amplification model ($F_{\text{S}}$), and calibration constant (*C*).

The source term (*F*_E_) isolates the effects of magnitude and stress parameter on the ground-motion amplitudes:

2 $$F_{\text{E}} = F_{\text{M}} + F_{\Delta \sigma },$$

where *F*_M_ represents the magnitude scaling term, ignoring near-distance-saturation effects, and $F_{\Delta \sigma }$ represents the stress parameter scaling term. The *F*_M_ term is a function of moment magnitude (**M**) and is defined using a hinged-quadratic functional form that follows an empirical form from data-rich regions (e.g., Boore et al. 2014):

3 $$F_{\text{M}} = \left\{ {\begin{array}{*{20}c} {e_{0} + e_{1} \left( {{\mathbf{M}} - {\mathbf{M}}_{\text{h}} } \right) + e_{2} \left( {{\mathbf{M}} - {\mathbf{M}}_{\text{h}} } \right)^{2} } & {{\mathbf{M}} \le {\mathbf{M}}_{\text{h}} } \\ {e_{0} + e_{3} \left( {{\mathbf{M}} - {\mathbf{M}}_{\text{h}} } \right)} & {{\mathbf{M}} > {\mathbf{M}}_{\text{h}} } \\ \end{array} } \right.$$

where the hinge magnitude, **M**_h_, and the model coefficients, *e*_0_, *e*_2_, and *e*_3_, are coefficients that are specified for each oscillator frequency (see “Appendix”).

High-frequency ground-motion amplitudes relative to low-frequency amplitudes are controlled by the stress parameter (Boore 2003). The stress adjustment term is defined as

4 $$F_{\Delta \sigma } = e_{\Delta \sigma } { \ln }\left( {\Delta \sigma /100} \right),$$

where *e*_Δ*σ*_ describes the rate of the ground-motion scaling with the stress parameter (Δ*σ*). The values of *e*_Δ*σ*_ as determined from the simulations have a variability in magnitude and frequency that is rather complicated, and the shape of the function differs depending on whether one is upscaling or downscaling the stress parameter. The shape can be described by a polynomial:

5 $$e_{\Delta \sigma } = \left\{ {\begin{array}{ll} s_{0} + s_{1} {\mathbf{M}} + s_{2} {\mathbf{M}}^{2} + s_{3} {\mathbf{M}}^{3} + s_{4} {\mathbf{M}}^{4} & \Delta \sigma \le 100 \;{\text{bar}} \\ s_{5} + s_{6} {\mathbf{M}} + s_{7} {\mathbf{M}}^{2} + s_{8} {\mathbf{M}}^{3} + s_{9} {\mathbf{M}}^{4} & \Delta \sigma > 100 \;{\text{bar}} \\ \end{array} } \right.$$

where *s*_0_ to *s*_9_ are frequency-dependent coefficients.

Geometrical spreading effects are modeled using an equivalent point-source distance metric:

6 $$R = \sqrt {D_{\text{rup}}^{2} + h^{2} },$$

where *h* is a pseudodepth term that accounts for distance saturation effects. The pseudodepth term is adopted from inversion results for active regions (Yenier and Atkinson 2015a), for which there are sufficient data to constrain such effects:

7 $$h = 10^{{ - 0.405 + 0.235{\mathbf{M}}}}.$$

It is important to note that, because we model geometric spreading using an equivalent point-source distance, the geometric spreading term implicitly includes the near-distance saturation effects attributable to finite-fault effects for large events. For small to moderate events, *D*_rup_ is approximately equal to the hypocentral distance (*D*_hypo_), and the geometric spreading is that of a classic point source.

The geometrical spreading function (*F*_Z_) is

8 $$F_{Z} = { \ln }\left( Z \right) + \left( {b_{3} + b_{4} {\mathbf{M}}} \right) {\text{ln}}\left( {R/R_{\text{ref}} } \right),$$

where *Z* represents the geometrical attenuation of Fourier amplitudes, whilst the multiplicative component, (*b*_3_ + *b*_4_**M**)ln(*R*/*R*_ref_), accounts for the change in the apparent attenuation that occurs when ground motions are modeled in the response spectral domain rather than the Fourier domain. *R*_ref_ is the reference effective distance, given as $R_{\text{ref}} = \sqrt {1 + h^{2} }$. *Z* is a hinged bilinear model that provides for a transition from direct-wave spreading to surface-wave spreading of reflected and refracted waves, beyond the critical distance for reflections from the Moho:

9 $$Z = \left\{ {\begin{array}{*{20}c} { R^{{b_{1} }} \quad R \le R_{\text{t}} } \\ { R_{\text{t}}^{{b_{1} }} \left( {R/R_{\text{t}} } \right)^{{b_{2} }}\quad R > R_{\text{t}} } \\ \end{array} } \right.$$

where *R*_t_ represents the transition distance (= 50 km), and *b*_1_ (= − 1.3) and *b*_2_ (= − 0.5) are the geometrical attenuation rates of Fourier amplitudes at *R* ≤ *R*_t_ and *R* > *R*_t_, respectively. Note that the coefficients describing geometric spreading and anelastic attenuation can be determined in ENA from empirical data for small to moderate earthquakes.

The site effects ($F_{\text{S}}$) are given relative to a reference site condition, in this case hard rock (travel-time weighted average shear-wave velocity over the top 30 m, *V*_S30_ ~ 2000 m/s); this is the site condition corresponding to most seismograph records in eastern Canada. The approach taken in Atkinson et al. (2015) was to use regression to determine site terms directly from the observations, along with the regional coefficients for attenuation.

The key attribute of the methodology behind the generic GMM is that most of the magnitude and distance scaling terms are fixed by previously calibrated simulation studies in data-rich regions, whilst a select few parameters—specifically the average stress parameter, anelastic attenuation, and calibration constant—are fine-tuned for the region of interest. In other words, we calibrate a well-behaved and validated generic model for a specific region of interest; the calibration can be accomplished using limited data on amplitude levels, site attributes, and attenuation.

**Fig. 2:**
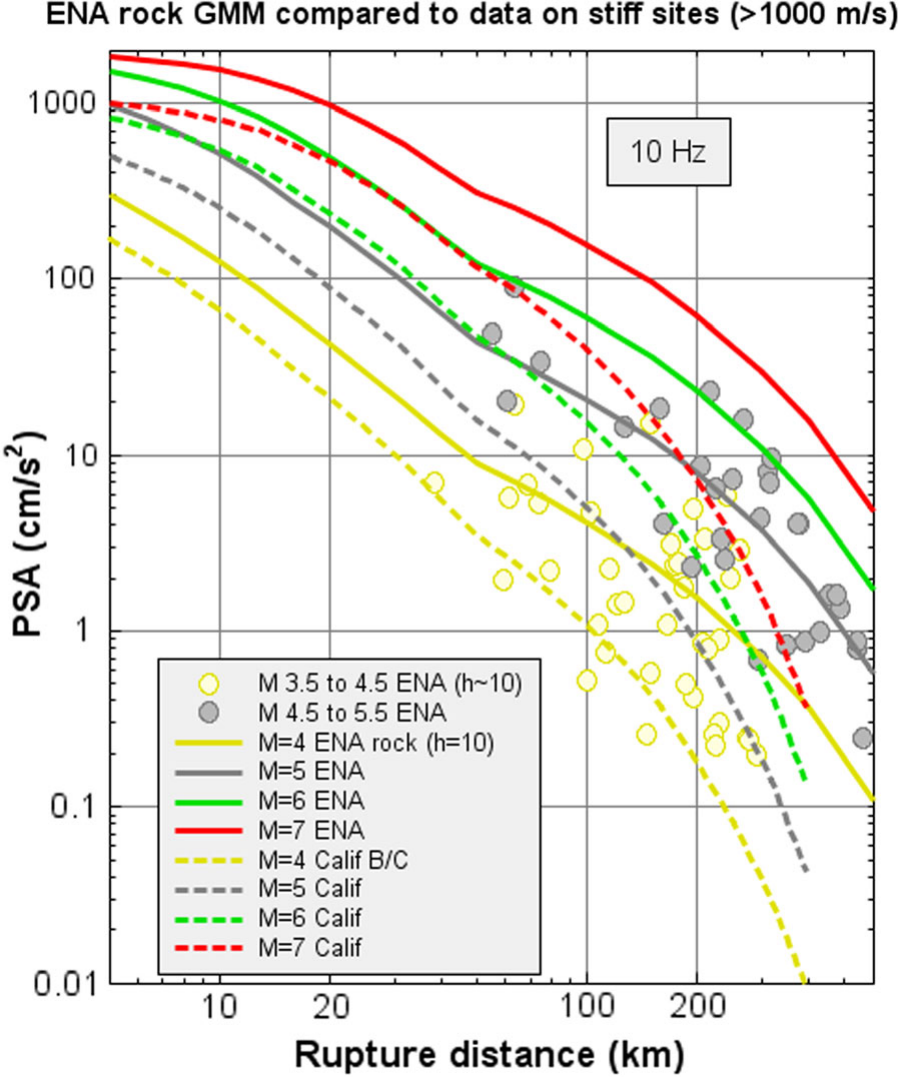
ENA GMM for hard rock site conditions (*V*_S30_ ≈ 2000 m/s) (solid lines) compared with eastern Canadian data recorded on stiff (*V*_S_ > 1000 m/s) and rock sites (*V*_S_ ~ 2000 m/s) (circles). California GMM for *B*/*C* sites based on the same model is shown for comparison, using dashed lines

The generic GMM developed for rock sites in eastern Canada is illustrated in Fig. 2 and compared with the corresponding functions for California. The comparison is made at high frequencies, for which rock amplitudes are significantly higher in ENA than in California due to a larger average value of stress parameter. At low frequencies (not shown), ENA and California amplitudes agree more closely. Note that the available data range for observations in ENA is limited, so for larger magnitudes the overall scaling behavior is effectively constrained by the underlying seismological model, which was calibrated for events in California of **M** 3.0–7.5.

Note that, because the generic GMM is based on an equivalent point-source concept, it will not adequately reproduce site-specific finite-fault attributes that would be important for sites that are very near to large potentially active faults. Such features could include directivity effects and strong coherent pulses, for example. Instead, the equivalent point source represents just the average of all such effects, via calibration to the California database. This representation is appropriate for most sites in ENA, for which the hazard is dominated by moderate events occurring on unknown faults within an areal source zone. More complex simulation models may be required for sites at which the hazard is influenced by specific nearby fault sources. Another model limitation is that finite-fault effects such as the near-distance saturation are assumed to be transferable from one region to another. This may not be completely true, since high-stress regions imply smaller faults than low-stress regions, and thus the near-distance saturation effects may be weaker in ENA than those observed in California. Such limitations of the equivalent point-source model with respect to the treatment of finite-fault effects are implicitly considered second-order effects, which we do not attempt to capture in the generic GMM approach.

Hassani and Atkinson (2018) further generalized the generic GMM to enhance its usefulness for a wider range of regions and site conditions, by including a new term in the GMM to account for the effects of the near-surface attenuation parameter (*κ*_0_) on the response spectral domain ground-motion amplitudes (PSA) as well as on the ground-motion peak amplitudes (PGA and PGV):

10 $$\ln \left( Y \right) = F_{\text{E}} + F_{{\kappa_{0} }} + F_{\text{Z}} + \gamma D_{\text{rup }} + F_{\text{S}} + C.$$

**Fig. 3:**
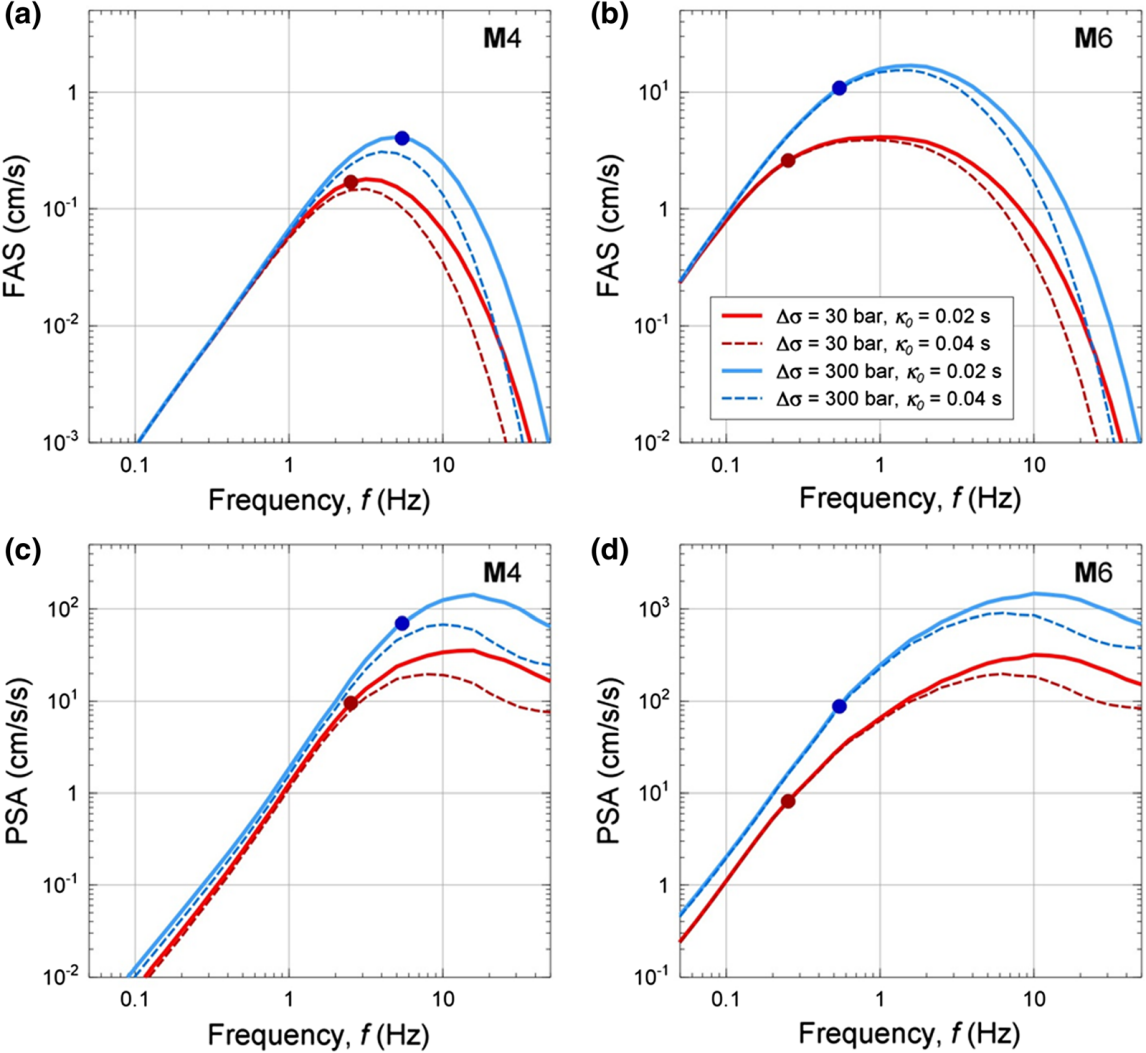
Illustration of the effects of stress parameter (values of 30 bar and 300 bar are shown) and kappa (values of 0.02 s and 0.04 s are shown) on ground-motion amplitudes in the Fourier domain (**a**, **b**) and response spectral domain (**c**, **d**), for **M **= 4 and **M** = 6 at *R* = 10 km. Dots show the corner frequency associated with the specified magnitudes and stress parameter values (from Yenier and Atkinson 2014)

The kappa term ($F_{{\kappa_{0} }}$) models the effects of near-surface high-frequency attenuation effects (*κ*_0_) (Anderson and Hough 1984; Van Houtte et al. 2011) in the response spectral domain. The interplay between the stress parameter and kappa is what controls the amplitudes of ground motions at high frequencies. This interplay is illustrated in Fig. 3 (see Boore 2003 for details).

The inclusion of a kappa term makes the GMM somewhat more complicated but facilitates adjustment of the *κ*_0_ value within the modified generic GMM to model a broader range of regions and reference site conditions. For further details of the use of this form, the reader is referred to Hassani and Atkinson (2018). Another advantage of having a *κ*_0_ term within the GMM is the ability to invert for the *κ*_0_ value using the response spectral amplitudes.

**Fig. 4:**
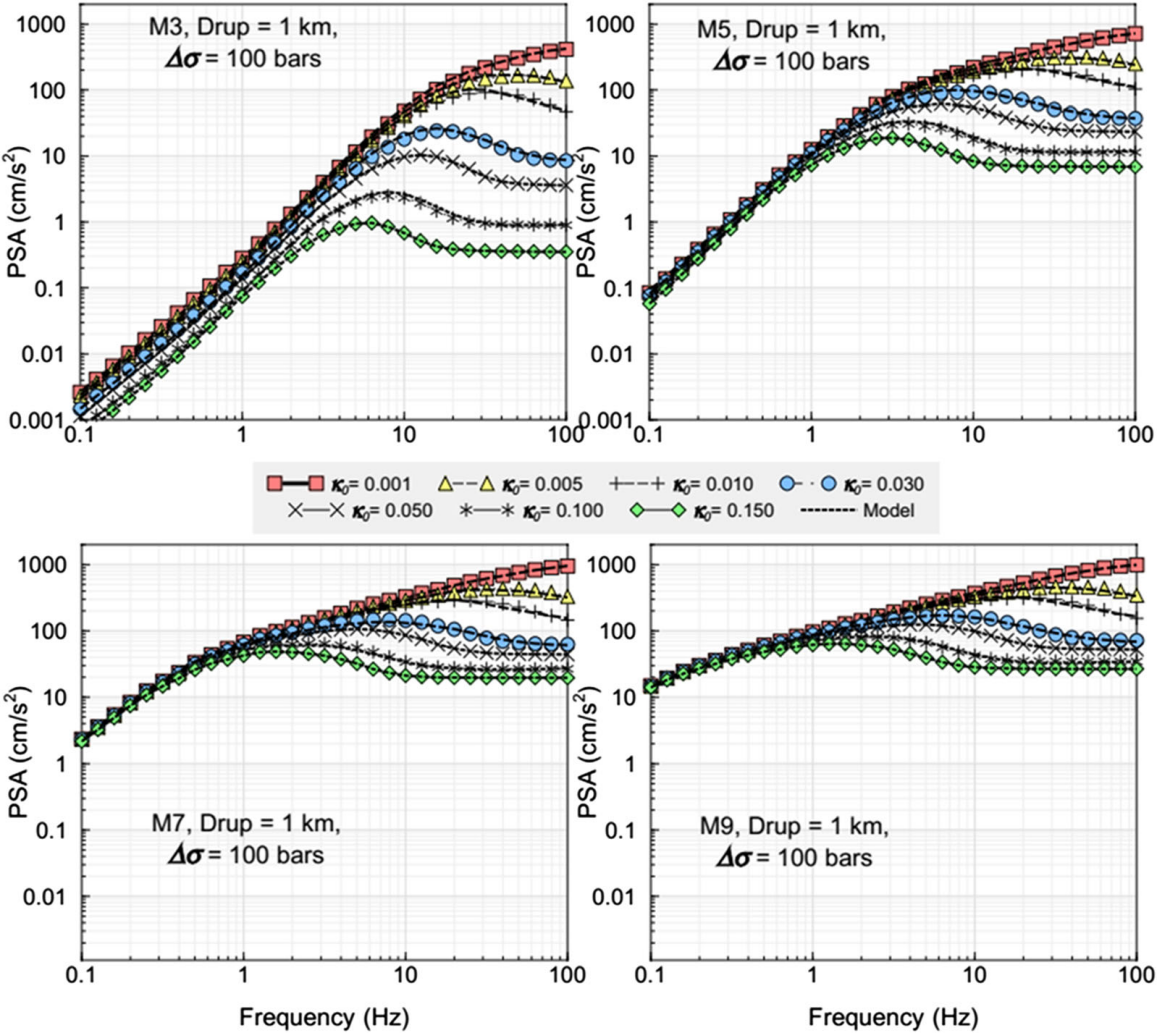
Response spectral amplitudes at 1 km for hard-rock sites from Hassani and Atkinson GMM model for **M** 3, 5, 7, and 9 for a stress parameter of 100 bar, showing influence of kappa (from Hassani and Atkinson 2018)

Figure 4 shows the response spectral amplitudes at near-source distance (*D*_rup_ = 1 km) for ∆*σ* = 100 bar and different *κ*_0_ values, for the adjustment model of Hassani and Atkinson (2018), over a wide range of magnitudes. This illustrates how maximum ground-motion amplitudes (before attenuation by path effects) are influenced by kappa in the response spectral domain. Effects can be pronounced at *f* > 10 Hz; For example, based on calculations with the model (not shown), for an event of **M **= 6 having a stress parameter of 300 bar, median 20-Hz PSA at 10 km would be ~ 490 cm/s^2^ for a very hard rock site with *κ*_0_ = 0.002. This scenario is the type of event that contributes significantly to hazard for nuclear sites in ENA situated on hard rock. However, it has been suggested that kappa values on some rock sites may be significantly higher than on others. For the same scenario event at a site for which *κ*_0_ = 0.01, the median PSA would be only ~ 330 cm/s^2^. Thus, kappa is an important high-frequency site parameter for rock sites.

The generic GMM model as presented in the foregoing is a useful way to encapsulate simple seismological models into a convenient functional form that facilitates the modeling of key source effects (**M**, stress parameter), path effects (geometric spreading and anelastic attenuation), and site effects (near-surface amplification and kappa), without the need to repeat simulations. It can be calibrated to limited regional ground-motion observations to provide a complete and robust GMM that follows scaling constraints in magnitude–distance space as established by empirical GMMs in data-rich regions. As such, it forms a practical and effective bridge between empirical and simulation-based modeling approaches.

## Aleatory Uncertainty

Seismic hazard is driven not only by median ground motions but also by their uncertainty. Uncertainty is, partly by convention, partitioned into components expressing random variability about the median (aleatory uncertainty) and uncertainty regarding the true median values (epistemic uncertainty) (Bommer and Scherbaum 2008; Strasser et al. 2009). These uncertainties imply that there is a significant probability of receiving ground motions much larger than those expressed by the median GMM. The aleatory uncertainty can be appreciated by inspection of Figs. 1 and 2, which show that amplitudes a factor of two or more above the median are not unusual.

The aleatory uncertainty is expressed by sigma, the standard deviation of residuals [defined as the ln(observed) – ln(predicted) amplitudes]. The total sigma can be partitioned into components that express between-event variability (*τ*) and within-event variability (*φ*). *τ* reflects the fact that some events are stronger than others due to their source attributes, such as a higher or lower stress parameter, whilst *φ* reflects deviations from the median attenuation curve within a single event. *φ* is sometimes further subdivided into components expressing the within-event variability component for a single station (*φ*_SS_) and the site-to-site variability (*φ*_S2S_); *φ*_S2S_ represents the systematic deviation of the ground motion at a specific site from the median event-corrected ground motion predicted by the GMM (in which only a general site-class model is included). The reader is referred to Al Atik et al. (2010) for details. Note that the values of the aleatory uncertainty components are themselves subject to epistemic uncertainty; that issue is not addressed here.

An interesting point is that *φ*_S2S_ and *τ* could both be considered largely epistemic in nature, as they represent systematic departures that may be predictable with improved knowledge. Specifically, *φ*_S2S_ is attributable to site-specific amplification, which can be measured relative to the predictions of a GMM for a generic site condition. In the case of *τ*, the hazard at a specific site may be dominated by a specific source having repeatable source attributes that could, at least in theory, be defined by site- and source-specific studies, leading to low between-event variability for the considered source. Atkinson (2006) showed that, by restricting a site-specific GMM to consider only earthquakes from a single source, the variability was significantly reduced (beyond that obtained by considering a single site).

There is strong motivation to reduce sigma to its lowest possible values at nuclear sites, because the value of sigma significantly impacts the PSHA results. For plants that are founded on hard rock, such as those in eastern Canada and some parts of the eastern USA, this can be accomplished by investing in seismic monitoring in the plant region, including in the plant vicinity, and focusing the GMM development on rock sites. In this approach, recorded seismographic data from regional earthquakes are used to calibrate the GMM, with the regression analysis including the derivation of the site-specific amplification term (as a function of frequency) for each site, relative to the GMM. To ensure stable site-specific amplifications, a minimum of five regional events (i.e., **M **> 3 within a few hundred km) should be recorded at each station. This will suffice to obtain the linear amplification for each site; the nonlinear component is typically determined separately based on either an empirical or analytical model (e.g., Harmon et al. 2018). An illustration of the approach is provided in Atkinson et al. (2015). They show that the total variability of the resulting GMM, which includes the site-specific amplification model at each station, is 0.50–0.58 ln units for events of small to moderate magnitude (**M** 3 to 6), recorded at distances to 500 km; this is significantly lower than the corresponding values for GMMs that do not model amplification on a site-specific basis (e.g., Goulet et al. 2017).

**Fig. 5:**
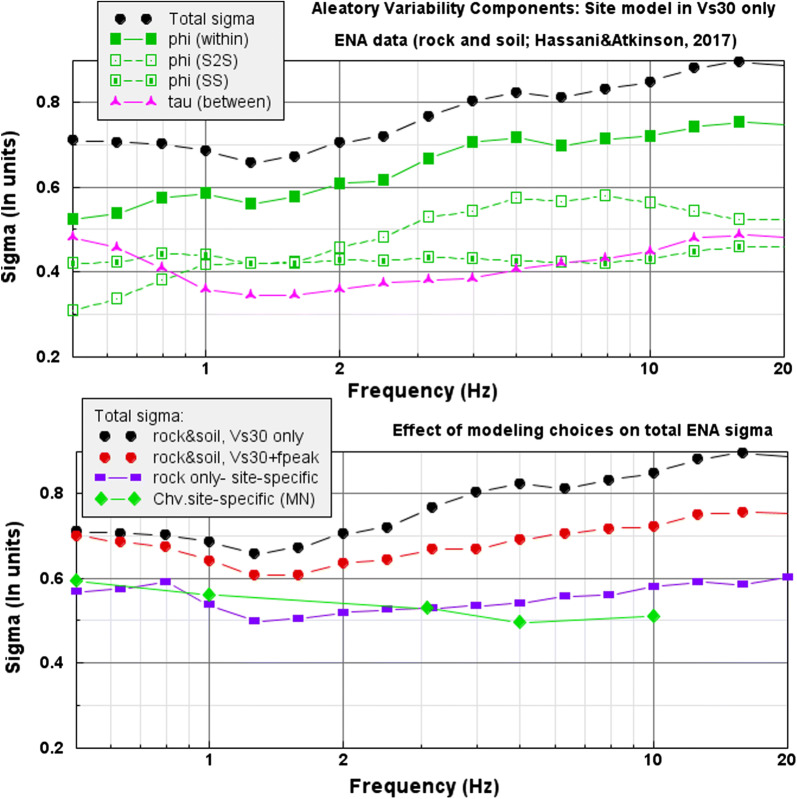
Top—components of aleatory variability (sigma) for ENA rock and soil data (from Hassani and Atkinson 2017) when *V*_S30_ is the only site variable, showing total sigma and its components (between-event *τ*, within-event *φ*, and its subcomponents *φ*_S2S_ and *φ*_SS_). Bottom—effect of GMM site-variable modeling choices for ENA, showing sigma from Hassani and Atkinson (2017) data if modeling rock and soil data with *V*_S30_ only (black), with both *V*_S30_ and *f*_peak_ (red), or using only rock data and removing site-specific response terms (purple squares). Green diamonds show sigma using site-specific GMM for Charlevoix modeled in MN (a high-frequency magnitude) (from Atkinson 2013)

By contrast to an approach that treats site response attributes directly within the GMM, inappropriate modeling of site effects will result in an inflated value of sigma. This is illustrated in Fig. 5, which was compiled using data on sigma from Hassani and Atkinson (2017) with respect to the rock GMM model for ENA as presented in this paper; some data on sigma for rock sites in the Charlevoix region relative to optimized GMMs from Atkinson (2013) are also shown. When all ENA data, including both rock and soil sites, are used to compute sigma, and we model site response in the GMM using only *V*_S30_ as a predictive variable, we attain high sigma values, about 0.8 ln units at high frequencies—which represents a factor of 2.2 in variability about the median for one standard deviation. If we improve the site model by including peak frequency of response for the site (*f*_peak_)—which in ENA is a more important site variable than *V*_S30_—then we reduce the sigma by about 0.1 ln units at high frequencies. Most of this reduction comes from the *φ*_S2S_ component for soil sites. If we consider only rock-like sites (those with *V*_S30_ > 1000 m/s), and model the site response at each specific station based on the seismographic data recorded at the site, the total sigma drops to values in the range of 0.5–0.6 ln units, or a factor of 1.7 about the median, again due largely to reduction of *φ*_S2S_. Finally, if the GMM uses a magnitude scale based on high-frequency amplitudes (e.g., Nuttli magnitude), instead of moment magnitude, we can reduce the *τ* component at higher frequencies (Atkinson 1995). As an example, Atkinson (2013) showed that total sigma for rock sites in the Charlevoix region, when modeled using Nuttli magnitude (MN), is ~ 0.5 ln units. Moreover, these sigma values are attained for small to moderate events, which typically have higher sigma than larger events, due to greater event-to-event variability in source parameters (e.g., Goulet et al. 2017).

The conclusion from the foregoing discussion is that sigma could be greatly reduced for nuclear sites on rock, resulting in lower calculated hazard. However, this reduction can only be achieved if a high-quality seismic monitoring network (e.g., broadband seismograph stations) is installed and operated over a period of years, and investment is also made in data analysis and targeted GMM development using the data. This has not been the approach taken in most parts of ENA to date. It should also be noted that, whilst reduction of sigma will lead to reductions in computed hazard for rock sites, and other sites with relatively low site amplification, the computed hazard may increase at sites where it is currently being underestimated by non-site-specific approaches.

## Epistemic Uncertainty

Epistemic uncertainty in median GMMs has often been modeled using alternative equations (typically those derived by various authors and approaches), with model weights in a PSHA logic tree being used to represent the relative confidence in each alternative. However, this is not necessarily the best way to model epistemic uncertainty in GMMs (Bommer and Scherbaum 2008; Atkinson 2011; Atkinson and Adams 2013; Atkinson et al. 2014). An alternative often used in site-specific studies is to define a representative or central-branch GMM, along with upper and lower variants that express uncertainty about the central model. This approach offers more flexibility in expressing uncertainty in knowledge of the correct median GMM. The representative equation approach also has significant practical utility, enabling a complex problem to be represented by a minimum number of branches for hazard calculations, which is efficient and transparent. A drawback is that a significant degree of judgment need be exercised regarding the selection of the central model and its upper and lower branches. However, such subjective judgments are equally important when using the alternative-GMM approach, as the selection and weighting of alternative models is also a process based on subjective judgment. To get around the drawbacks of both the representative suite and alternative GMM approaches, a more sophisticated and objective approach to representing model alternatives, based on Sammon’s mapping of predicted amplitudes in higher-order dimensions, has been used in some projects, such as the NGA-East project (Goulet et al. 2017). This is a powerful approach but not easy to implement; it is also cumbersome to adjust the model on a site-specific basis as more information is obtained.

To some extent, the details of the method used to represent epistemic uncertainty may not be of critical importance. Sensitivity tests indicate that it is the range covered by the GMM models and their relative weights that are important to the PSHA results, not the mechanics of how they are treated (Atkinson and Adams 2013). An additional consideration is that the division of ground-motion uncertainty into its epistemic and aleatory components is ambiguous and nonunique, because some factors of the total uncertainty could be cast into either component (Strasser et al. 2009). In contemplating the epistemic versus aleatory subdivision, a factor to consider is that, whilst GMMs cover a range of possible magnitude–distance scenarios, the actual “design event” that an individual structure may be required to withstand is really a single unknown future event that will have specific source, path, and site attributes, at some sigma level. This is because large potentially damaging earthquakes are rare events, and most structures will be expected to withstand only one such event in their design life. Moreover, for a nuclear plant, there would be inspection and repair immediately following any strong event and a reset of the facility’s capacity. We do not know in advance the specifics of the event that the plant may experience, but we can model this uncertainty within the context of PSHA in a few ways. The simplest, assuming we also wish to use site-targeted aleatory uncertainty as described in the previous section, is to use the representative suite approach to define the epistemic uncertainty about the central branch GMM. In concept, the interevent components of uncertainty (most of the *τ* component of aleatory variability) could be largely modeled as epistemic, reflecting uncertainty in the source characteristics that are expected to be realized in future events through the alternative GMM branches. In this case, the aleatory uncertainty would represent just the variability of observations about a median event-specific prediction equation for a single station (i.e., *φ*_SS_). Considering the variability components of Fig. 5, careful source-driven modeling of epistemic uncertainty might reduce the aleatory component to the range of 0.4–0.5 ln units.

Such an approach presupposes that we can model epistemic uncertainty in ground motions from future events through definition of the distribution of earthquake source, path, and site parameters of the GMM model. The generic GMM approach outlined here is a practical way to implement such distributions in Monte Carlo PSHA software, in which simulated earthquake catalogs and their generated ground motions at a site are used to calculate the ground-motion distribution at a site (Musson 1999; Assatourians and Atkinson 2013). For each simulated earthquake magnitude and location, we would draw (by Monte Carlo, from a defined distribution) a value of stress parameter and a value of total attenuation from source to site (frequency dependent, including geometric spreading and anelastic effects). The aleatory uncertainty for calculations at a specified site (with known response characteristics from network observations) would be that attributable to *φ*_SS_. This approach would allow PSHA to be more site specific in its application of epistemic and aleatory uncertainties.

## Concluding Remarks

This paper has dealt with the use of conventional GMMs to define ground motion and its epistemic and aleatory uncertainty, within the context of contemporary PSHA methodology. In the longer term, a truly site-specific PHSA would be based on simulations that fully consider the source, path, and site attributes that govern ground motions for all potential future earthquake scenarios, eliminating the need for GMMs altogether (e.g., Atkinson 2012). However, such an approach will require substantial further improvement of our knowledge of earthquake source, path, and site processes. Until such advances can be achieved, GMMs that bridge seismological and empirical approaches are an effective tool to represent the distribution of ground motion and its uncertainty in seismic hazard assessment.

## Appendix: Coefficient Tables for ENA Rock GMM of Atkinson et al. (2015)

**Table:**

Freq. (Hz)	*C*	**M**_h_	*e*_0_	*e*_1_	*e*_2_	*e*_3_	*b*_3_	*b*_4_	*γ*
0.10	− 0.250	7.45	− 0.2763	1.9720	− 0.0463	1.7230	− 0.6196	0.08420	− 0.00130
0.20	− 0.250	6.85	− 0.0792	1.9800	− 0.0621	1.5850	− 0.5800	0.07896	− 0.00130
0.33	− 0.250	6.65	0.5156	1.9080	− 0.0898	1.4180	− 0.5129	0.06761	− 0.00130
0.50	− 0.250	6.65	1.2510	1.7480	− 0.1316	1.1920	− 0.4353	0.05361	− 0.00130
0.63	− 0.445	6.75	1.7530	1.5640	− 0.1678	1.0540	− 0.3849	0.04430	− 0.00130
0.77	− 0.605	6.75	2.0110	1.3860	− 0.2057	1.0000	− 0.3503	0.03777	− 0.00130
1.00	− 0.770	6.45	1.9860	1.3400	− 0.2456	0.9829	− 0.2974	0.02765	− 0.00130
1.25	− 0.878	6.85	2.6640	0.9053	− 0.2888	0.8944	− 0.2523	0.01906	− 0.00130
1.54	− 0.951	6.60	2.6170	0.8758	− 0.3160	0.9251	− 0.2277	0.01371	− 0.00130
2.00	− 1.007	6.25	2.5440	0.8856	− 0.3486	0.9182	− 0.2079	0.00854	− 0.00130
2.50	− 1.022	6.15	2.6740	0.8501	− 0.3469	0.8966	− 0.1927	0.00485	− 0.00173
3.33	− 0.997	5.85	2.6350	0.8471	− 0.3631	0.8763	− 0.2117	0.00516	− 0.00231
4.00	− 0.956	5.60	2.5170	0.8671	− 0.3775	0.8785	− 0.2429	0.00921	− 0.00267
5.00	− 0.878	5.45	2.5490	0.8194	− 0.3860	0.8426	− 0.2868	0.01376	− 0.00312
6.25	− 0.770	5.25	2.4660	0.8088	− 0.3871	0.8407	− 0.3265	0.01918	− 0.00357
7.69	− 0.643	5.35	2.6410	0.7346	− 0.3321	0.8116	− 0.3551	0.02224	− 0.00398
10.0	− 0.600	5.45	2.7770	0.7118	− 0.2619	0.7941	− 0.3774	0.02472	− 0.00451
12.5	− 0.600	5.20	2.5760	0.7651	− 0.2434	0.7865	− 0.4210	0.03071	− 0.00495
15.4	− 0.600	5.75	2.9970	0.6842	− 0.1547	0.7553	− 0.4665	0.03640	− 0.00537
20.0	− 0.600	5.35	2.5590	0.7193	− 0.1636	0.7545	− 0.5096	0.04279	− 0.00550
50.0	− 0.600	5.90	2.3780	0.6999	− 0.1066	0.7488	− 0.6377	0.06251	− 0.00550
PGA	− 0.380	5.85	2.2160	0.6859	− 0.1392	0.7656	− 0.6187	0.06029	− 0.00490
PGV	− 0.740	5.90	5.9600	1.0300	− 0.1651	1.0790	− 0.5785	0.05737	− 0.00290

Freq. (Hz)	*s*_0_	*s*_1_	*s*_2_	*s*_3_	*s*_4_	*s*_5_	*s*_6_	*s*_7_	*s*_8_	*s*_9_
0.10	− 2.1940	1.5110	− 0.2871	0.01534	0.00024	0.2684	− 0.3862	0.2169	− 0.03967	0.00230
0.20	− 6.3880	5.0830	− 1.3810	0.15760	− 0.00636	− 1.3770	0.9093	− 0.1422	0.00132	0.00071
0.33	− 7.9800	6.6430	− 1.9240	0.23660	− 0.01039	− 4.1800	3.3170	− 0.8862	0.09888	− 0.00385
0.50	− 6.6420	5.7670	− 1.7420	0.22410	− 0.01028	− 6.0100	4.9850	− 1.4330	0.17480	− 0.00759
0.63	− 5.2640	4.7380	− 1.4760	0.19630	− 0.00928	− 5.8800	4.9780	− 1.4650	0.18320	− 0.00816
0.77	− 2.5080	2.5230	− 0.8446	0.12050	− 0.00602	− 5.4940	4.7660	− 1.4390	0.18490	− 0.00846
1.00	1.0660	− 0.4552	0.0374	0.01033	− 0.00108	− 4.4730	4.0510	− 1.2740	0.17100	− 0.00814
1.25	2.4040	− 1.6520	0.4088	− 0.03710	0.00105	− 2.1240	2.1520	− 0.7301	0.10530	− 0.00529
1.54	3.6450	− 2.8220	0.7932	− 0.08926	0.00356	− 0.6671	0.9277	− 0.3708	0.06183	− 0.00343
2.00	3.9560	− 3.2880	0.9885	− 0.11960	0.00514	0.8555	− 0.4528	0.0646	0.00522	− 0.00083
2.50	2.0180	− 1.8570	0.6117	− 0.07674	0.00334	2.4220	− 1.9380	0.5558	− 0.06174	0.00239
3.33	− 0.3182	− 0.1386	0.1704	− 0.02850	0.00142	2.2450	− 2.0030	0.6326	− 0.07699	0.00327
4.00	− 1.7670	0.9826	− 0.1314	0.00600	− 0.00001	1.7800	− 1.7670	0.6066	− 0.07834	0.00350
5.00	− 2.7070	1.7290	− 0.3302	0.02816	− 0.00091	0.8197	− 1.0830	0.4395	− 0.06105	0.00285
6.25	− 3.9650	2.8200	− 0.6499	0.06720	− 0.00261	− 0.1997	− 0.3370	0.2570	− 0.04252	0.00218
7.69	− 4.1740	3.0920	− 0.7438	0.07982	− 0.00321	− 1.3840	0.6264	− 0.0116	− 0.01092	0.00083
10.0	− 4.0510	3.1000	− 0.7625	0.08328	− 0.00339	− 2.4520	1.5690	− 0.2890	0.02300	− 0.00066
12.5	− 3.6850	2.9620	− 0.7510	0.08421	− 0.00351	− 3.1390	2.1770	− 0.4674	0.04466	− 0.00160
15.4	− 2.2250	1.9480	− 0.4900	0.05486	− 0.00229	− 3.9600	2.8710	− 0.6675	0.06883	− 0.00265
20.0	− 0.9628	0.9826	− 0.2156	0.02080	− 0.00074	− 4.2280	3.2930	− 0.8316	0.09303	− 0.00387
50.0	− 1.1600	1.2740	− 0.3344	0.03911	− 0.00170	− 1.2720	1.2540	− 0.3171	0.03624	− 0.00155
PGA	− 2.1320	1.9370	− 0.5040	0.05824	− 0.00250	− 1.4440	1.2350	− 0.2851	0.03021	− 0.00122
PGV	− 2.2460	1.9510	− 0.5181	0.06139	− 0.00273	− 1.7580	1.3790	− 0.3256	0.03500	− 0.00143
